# Drugs Targeting the A3 Adenosine Receptor: Human Clinical Study Data

**DOI:** 10.3390/molecules27123680

**Published:** 2022-06-08

**Authors:** Pnina Fishman

**Affiliations:** Can-Fite BioPharma Ltd., 10 Bareket St., Petah Tikva 49170, Israel; Pnina@canfite.co.il; Tel.: +972-3-3241114

**Keywords:** A3AR, agonist, anti-fibrosis, anti-inflammatory, liver cancer, namodenoson, non-alcoholic steatohepatitis, piclidenoson, psoriasis

## Abstract

The A3 adenosine receptor (A3AR) is overexpressed in pathological human cells. Piclidenoson and namodenoson are A3AR agonists with high affinity and selectivity to A3AR. Both induce apoptosis of cancer and inflammatory cells via a molecular mechanism entailing deregulation of the Wnt and the NF-κB signaling pathways. Our company conducted phase I studies showing the safety of these 2 molecules. In the phase II studies in psoriasis patients, piclidenoson was safe and demonstrated efficacy manifested in significant improvements in skin lesions. Namodenoson is currently being developed to treat liver cancer, where prolonged overall survival was observed in patients with advanced liver disease and a Child–Pugh B score of 7. A pivotal phase III study in this patient population has been approved by the FDA and the EMA and is currently underway. Namodenoson is also being developed to treat non-alcoholic steatohepatitis (NASH). A Phase IIa study has been successfully concluded and showed that namodenoson has anti-inflammatory, anti-fibrosis, and anti-steatosis effects. A phase IIb study in NASH is currently enrolling patients. In conclusion, A3AR agonists are promising drug candidates in advanced stages of clinical development and demonstrate safety and efficacy in their targeted indications.

## 1. Introduction

The A3 adenosine receptor (A3AR) has been positioned as a target for combatting inflammation and cancer over the last 2 decades [1,2]. The target, Gi protein-coupled A3AR, is highly expressed in inflammatory and cancer cells [2,3,4,5]. Interestingly, high A3AR expression levels are also found in peripheral blood mononuclear cells (PBMCs) of patients with inflammatory diseases and cancer, reflecting A3AR expression in pathological remote sites. Solid tumor cells, including breast, colon, small cell lung and pancreatic carcinoma, and melanoma, highly express A3AR compared to normal adjacent tissue cells. A3AR is also expressed in inflammatory cells such as synoviocytes derived from patients with rheumatoid arthritis, skin biopsies, and PBMCs from psoriasis and Crohn’s disease patients [6,7,8,9,10].

Targeting the receptor with synthetic and highly selective A3AR agonists and allosteric modulators induces anti-inflammatory and anti-cancer effects [4,11,12,13,14,15,16].

## 2. A3AR Agonists: Piclidenoson and Namodenoson

**Figure 1:**
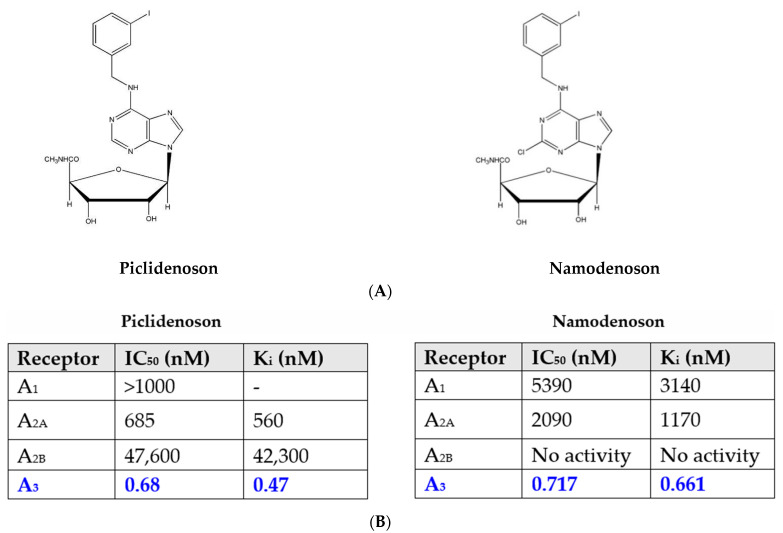
(**A**) Chemical structure of piclidenoson and namodenoson. (**B**) Affinity values of piclidenoson and namodenoson to the different adenosine receptors.

Piclidenoson (CF101, known generically as IB-MECA; molecular weight, 510.29 Da) and namodenoson (CF102, known generically as Cl-IB-MECA; molecular weight, 544.73 Da) are small, water-insoluble, orally bioavailable A3AR agonists. Both drugs are nucleoside derivatives, and their half-life time is 9 and 12 h, respectively. Their chemical structure is depicted in Figure 1.

### 2.1. Piclidenoson and Namodenoson: Pharmacological Activity

The activity of piclidenoson as an orally administered, anti-inflammatory and anti-cancer agent has been tested in several experimental models of inflammatory bowel disease, arthritis, osteoarthritis, uveitis, and non-alcoholic steatohepatitis (NASH) and cytokine release syndrome [16,17,18,19,20,21,22]. In all models, oral administration of piclidenoson induced a robust reduction in the inflammatory response as measured by clinical and histological disease scores. The anti-cancer activity was demonstrated in animal models of prostate, melanoma, colon, hepatocellular, and breast carcinoma [11,12,14,23,24].

In addition, A3AR agonists mediate a differential effect on pathological and normal cells. Thus, piclidenoson and namodenoson induce apoptosis in inflammatory and cancer cells, whereas normal cells are refractory to the effect of these drugs or are affected positively by the drug [25]. In cancer cells, where there is an over-expression of the receptor, treatment with an A3AR agonist leads to inhibition of regulatory proteins such as NF-κB, followed by tumor growth inhibition [23,24]. In normal bone marrow-derived cells, upon binding of the agonist, an up-regulation of NF-κB with a subsequent increase in the level of the cytokine granulocyte colony-stimulating factor (G-CSF) takes place and induces the proliferation of normal progenitor cells with an increase in the number of white blood cells and neutrophils, yielding a chemoprotective effect [26,27]. The most prominent example involves the anti-cancer and anti-inflammatory effect of namodenoson on pathological liver cells alongside its hepatoprotective effects on normal liver cells. This differential effect attributes to the good safety profile of the A3AR agonists [28,29].

### 2.2. Piclidenoson and Namodenoson: Mechanism of Action

**Figure 2:**
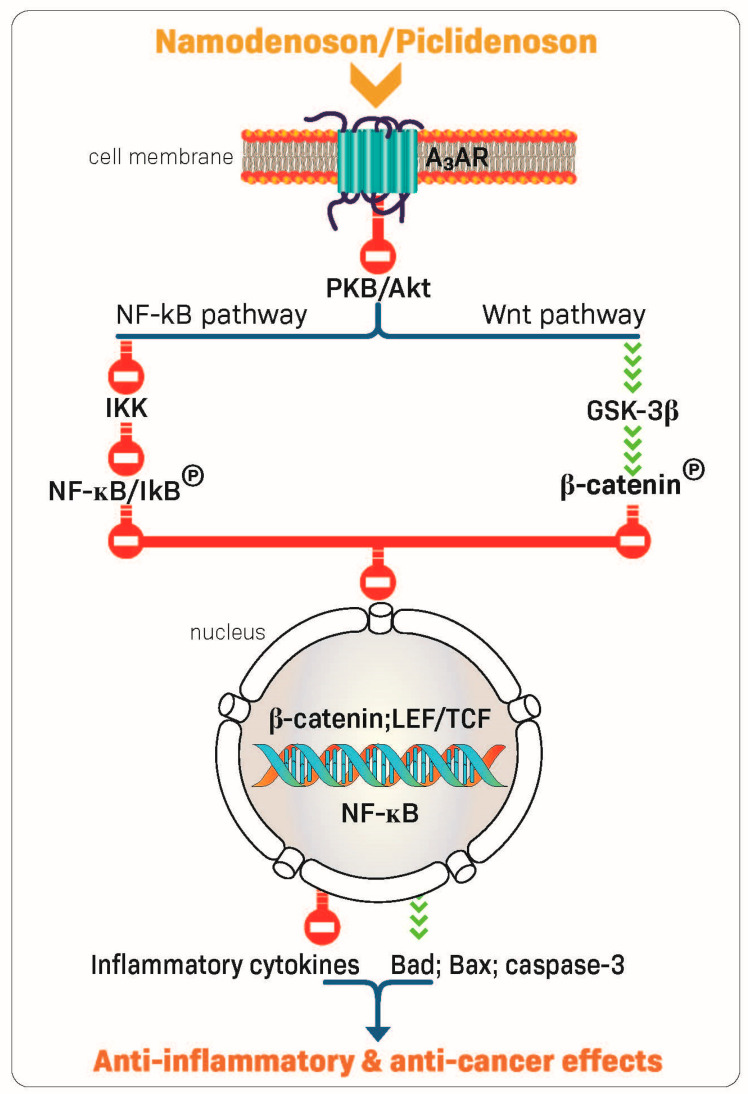
Mechanism of action of piclidenoson and namodenoson.

The robust anti-inflammatory and anti-cancer effects of piclidenoson and namodenoson are mediated via their ability to inhibit the production of inflammatory cytokines such as tumor necrosis factor (TNF)-α, interleukin (IL)-12, interferon-ɣ, IL-17, and IL-23 [22,30,31,32]. In addition, A3AR modulation of key signaling proteins, such as PI3K, GSK-3β, PKA, PKB/Akt, IKK, and β-catenin, was shown to lead to deregulation of the NF-κB and the Wnt/β-catenin signaling pathways. This chain of events eventually induces apoptosis of inflammatory and cancer cells, resulting in anti-inflammatory and anti-cancer effects (Figure 2) [23,33,34,35,36]. These mechanistic studies laid the foundation for the clinical development of piclidenoson and namodenoson.

## 3. Piclidenoson: Clinical Development

### 3.1. Piclidenoson in Rheumatoid Arthritis (RA)

RA is a chronic, progressive, and disabling autoimmune disease characterized by inflammation of the joints, destruction of cartilage, and erosion of the bone. RA can make performing activities of daily living challenging and can also impact the ability to fulfill social roles and obligations. The treatments recommended by both the American College of Rheumatology (ACR) and the European Alliance of Associations for Rheumatology (EULAR) include disease-modifying antirheumatic drugs (DMARDs), such as conventional synthetic DMARDs (e.g., methotrexate (MTX)), biologic DMARDs (e.g., TNF-α inhibitors), and targeted synthetic DMARDs (e.g., JAK inhibitors) as well as glucocorticoids. Notably, these drugs are associated with adverse events, and therefore, the need exists for a new approach in anti-RA therapy [37].

The definition of response to a given drug by the ACR is scored as a percentage improvement, comparing disease activity at two discrete time points (usually a baseline vs. end of treatment). ACR20 is ≥20% improvement; ACR50 is ≥50% improvement; and ACR70 is ≥70% improvement. The ACR response criteria measures improvement in tender or swollen joint counts and improvement in at least three of the following parameters: global patient assessment of disease activity, global physician assessment of disease activity, patient pain scale, disability/functional questionnaire (Health Assessment Questionnaire Disability Index) and acute phase reactant (erythrocyte sedimentation rate [ESR] or C-reactive protein) [38]. At the same time, the definition of EULAR recommends low disease activity (LDA) as a response criterion [39].

#### 3.1.1. Piclidenoson in RA: Phase IIa Study

In the first clinical study in RA, patients were randomized to receive piclidenoson in one of 3 doses: 0.1 mg, 1 mg, or 4 mg. The study was double-blinded with respect to the piclidenoson dose group. Piclidenoson was given as a monotherapy and administered twice daily for 12 weeks. The primary endpoint was the percentages of RA patients who met the ACR20, ACR50, and ACR70, criteria. The highest response rate was observed with 1.0 mg piclidenoson, and lower responses were found with 0.1 and 4.0 mg doses. At 12 weeks, 55.6%, 33.3%, and 11.5% of the patients receiving 1.0 mg piclidenoson achieved ACR20, ACR50 and ACR70 responses, respectively. Piclidenoson was safe and well-tolerated at all 3 doses [9].

#### 3.1.2. Piclidenoson in RA: Phase IIb Studies

Two phase IIb studies investigated piclidenoson in combination with MTX vs. MTX alone for the treatment of RA. One study included 230 patients (NCT00556894) and the other included 252 patients (NCT00280917). In both studies, at 12 weeks, no significantly better ACR20 response rate was observed for piclidenoson plus MTX vs. MTX alone. Therefore, both studies did not reach the primary endpoint. A cross-study analysis revealed that at baseline, A3AR was highly expressed in the PBMCs derived from the phase IIa patient population. In contrast, only low receptor expression was found in the two-phase IIb patient populations, as all patients in these phase IIb studies were pre-treated with MTX. Thus, pre-treatment with MTX reduced A3AR expression and diminished the ability of piclidenoson to evoke treatment effects. It is well established that the anti-inflammatory effect of MTX is mediated via the A2A and the A3 adenosine receptors. Therefore, patients who did not respond to MTX most likely had low A2 and A3 adenosine receptor levels and thus did not respond to piclidenoson [3].

An additional phase IIb study investigated piclidenoson as a monotherapy (NCT01034306). This was a 12-week, placebo-controlled study involving 79 patients in 2 arms (twice-daily monotherapy dose of 1 mg piclidenoson vs. placebo) [3]. The study met the primary efficacy endpoint and showed statistically significant improvement vs. placebo in reducing signs and symptoms of RA, as measured by the ACR20 response rates. The ACR20 response rate in the piclidenoson arm was 49% vs. 25% in the placebo arm (*p* = 0.035). The ACR50 response rates were 19% and 9%, respectively, and the ACR70 response rates were 11% and 3%, respectively. The study was not designed to show statistical significance in the ACR50 and ACR70 rates. The effect of piclidenoson was linear and cumulative during the study period [40], corroborating the effect found in the earlier RA as well as psoriasis phase II studies and showing that it takes time for the drug to induce its anti-inflammatory effect.

Notably, a subgroup analysis of 16 treatment-naïve patients (8 in the piclidenoson group and 8 in the placebo group) with no prior systemic therapy showed a dramatic increase in the response for piclidenoson vs. placebo. In these patients, at 12 weeks, the ACR20 rate was 75% in the piclidenoson arm vs. 37.5% in the placebo arm. The effect of piclidenoson in treatment-naïve patients was even more pronounced for ACR50 and ACR70. ACR50 rates were 50% and 10%, respectively, and ACR70 rates were 50% and 0%, respectively. We hypothesize that in treatment-naïve patients, A3AR is highly expressed.

Piclidenoson was very well-tolerated with no evidence of immunosuppression. In the piclidenoson plus MTX combination studies, no single adverse event was reported in more than one patient receiving piclidenoson, and in the monotherapy study, no severe treatment-emergent adverse events (TEAEs) were reported [40].

#### 3.1.3. Piclidenoson in RA: Phase III Study

Based on the phase IIb study data, a randomized, double-blind, controlled, parallel-group phase III study was conducted. It enrolled patients with clinically active RA who were MTX-naïve. Eligible patients were randomized to 4 groups in a 2:2:2:1 ratio: piclidenoson 1 mg; piclidenoson 2 mg; MTX (active comparator); and placebo. The primary efficacy endpoint was the non-inferiority of piclidenoson administered for 12 weeks vs. oral MTX, as assessed by the proportion of patients achieving a disease activity score of LDA. Additional analyses of the primary efficacy parameter included comparing each dose of piclidenoson to placebo at week 12. Secondary endpoints included comparing response rates (ACR20, ACR50, and ACR70) between piclidenoson (each dose) and MTX or placebo. The study was designed to enroll a total of 525 patients.

Due to the SARS-CoV-2 pandemic, enrollment was paused, and an interim analysis was performed after 50% of participants reached their week 12 visit evaluation. Piclidenoson was safe and very well tolerated. Although the efficacy of piclidenoson was significantly superior to that of a placebo, the study did not meet its primary endpoint since non-inferiority vs. MTX was not demonstrated. Results from the per-protocol population did not support the non-inferiority of piclidenoson 1 mg or 2 mg vs. MTX (one-sided analysis: *p* = 0.192 and *p* = 0.107, respectively). The results did indicate that piclidenoson 2 mg and MTX were both superior to placebo (*p* = 0.039 and *p* = 0.004, respectively). Analysis based on the intent-to-treat population indicated that neither piclidenoson 1 mg nor 2 mg were superior to placebo (two-sided analysis, *p* = 0.500 and *p* = 1.00, respectively); however, MTX was superior to placebo (two-sided analysis; *p* = 0.005) [41].

At weeks 12 and 24, ACR20, ACR50 and ACR70 response rates were overall similar between the piclidenoson 1 mg (which was more effective than the 2 mg dose) and the MTX arms.

Piclidenoson was safe in this population; however, since the endpoint was not met, the clinical development program in RA was terminated. Although the drug was safe, the efficacy, which with respect to ACR20, was inferior to MTX, did not justify the continuation of this clinical application in the two dosages used in the current study.

### 3.2. Piclidenoson in Psoriasis

Psoriasis is a chronic inflammatory systemic disease of the skin affecting 2–3% of the world population. It is characterized by hyperproliferation of keratinocytes and increased pro-inflammatory cytokines and chemokines in the psoriatic skin lesion, which attracts immune cells. The ability of keratinocytes to resist apoptosis plays an important role in disease pathogenesis. Furthermore, keratinocytes produce inflammatory cytokines such as TNF-α, IL-23, and IL-17 together with infiltrated immune cells, which trigger the vicious cycles of the disease [42]. Preclinical pharmacology studies demonstrated that piclidenoson inhibited the proliferation of human keratinocytes (HaCat cells). High A3AR expression levels were found in a skin biopsy and PBMCs from psoriasis patients compared to healthy subjects. Piclidenoson inhibited the proliferation of HaCat cells through deregulation of the NF-κB signaling pathway, leading to a decrease in IL-17 and IL-23 expression levels. This effect was counteracted by the specific antagonist MRS 1523. A3AR overexpression in the skin and PBMCs of psoriasis patients may be used as a target to inhibit pathological cell proliferation and the production of IL-17 and IL-23 [23].

The efficacy criteria in psoriasis are the Psoriasis Area and Severity Index (PASI) score and Physician’s Global Assessment (PGA) score. The PASI measures the average redness, thickness, and scaliness of the lesions (each graded on a 0–4 scale), weighted by the area of involvement. The PGA can be used for extensive disease as well as localised plaques. There are two primary forms: a static form, which measures the physician’s impression of the disease at a single point, and a dynamic form, in which the physician assesses the global improvement from baseline. Because the latter requires the dubious assumption that physicians can remember the severity of psoriasis at baseline throughout the trial, the static PGA has become the standard. The latter has many variations, including 5, 6, or 7-point scoring ranging from ‘clear’ to ‘severe’ [43].

#### 3.2.1. Piclidenoson in Psoriasis: Phase II Study

**Figure 3:**
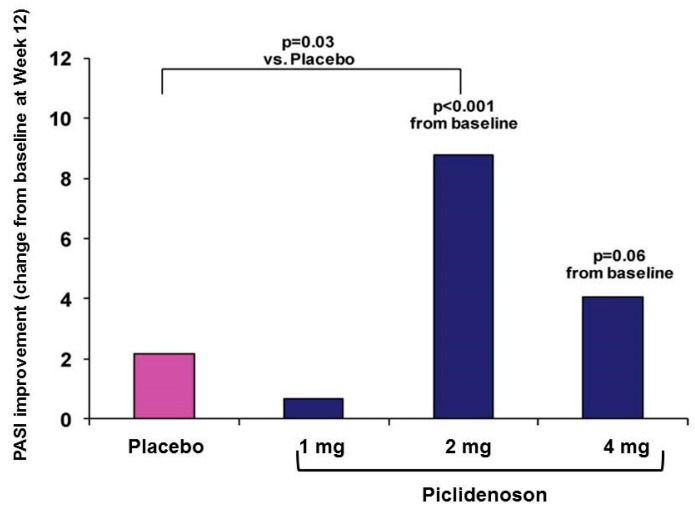
Change from baseline in PASI score by piclidenoson dose at week 12 [40].

The first phase II study investigating piclidenoson in psoriasis was an exploratory randomized clinical trial in patients with moderate-to-severe plaque-type psoriasis. This multicenter, randomized, double-blind, dose-ranging, placebo-controlled study involved 75 patients. Patients were treated with piclidenoson (1, 2, or 4 mg) or placebo, administered orally twice daily for 12 weeks. Safety and change from baseline in PASI and PGA scores over 12 weeks were assessed [40]. Analysis of the mean change from baseline in PASI score at week 12 revealed a statistically significant difference between the 2 mg piclidenoson-treated group and the placebo group (*p* < 0.001 vs. baseline and *p* = 0.031 vs. placebo). The 4 mg piclidenoson dose treatment resulted in reduced improvement than the 2 mg dose, and no therapeutic effect was observed with the 1 mg piclidenoson dose (Figure 3). Furthermore, in the 2 mg piclidenoson-treated group, a progressive improvement in the mean change from baseline in the PASI score throughout the study period was observed (week 2: 1.64 ± 0.9; week 4: 3.76 ± 1.9; week 8: 6.22 ± 1.9; week 12: 8.77 ± 2.1), with a statistically significant difference from placebo at weeks 8 and 12 (*p* = 0.047 and *p* = 0.031, respectively). The percentage of patients presenting only slight or no clinical signs (PGA score 0–1) increased throughout the study period in the 2 mg piclidenoson-treated group [44].

Notably, this bell-shaped dose-response (e.g., highest response with the intermediate dose) is a typical response generated by piclidenoson, observed in both in vitro and in vivo studies. Such a bell-shaped response was also observed with other agonists that target Gi protein-associated cell surface receptors. This phenomenon is explained in the literature by an increase in agonist concentration that may lead to receptor desensitization, resulting in a reduced response to the given agonist.

Piclidenoson was safe and well-tolerated. No drug-related serious adverse events were reported throughout the study period [44].

#### 3.2.2. Piclidenoson in Psoriasis: Phase II/III Study

A phase II/III study in psoriasis was initiated following the aforementioned phase II study. This was a randomized, double-blind, placebo-controlled, dose-finding study investigating the efficacy and safety of daily piclidenoson administered orally in patients with moderate-to-severe plaque psoriasis (NCT01265667). The study failed to achieve its primary endpoint of a statistically significant improvement in PASI-75 rate (75% improvement in PASI score from baseline) relative to placebo after 12 weeks [45].

Further analysis of the data revealed that by 32 weeks of treatment with piclidenoson, 33% of the patients achieved PASI-75. The mean percent of improvement in PASI score was 57% (*p* < 0.001 compared to baseline). There was a statistically significant cumulative and linear improvement during weeks 16 to 32. Most significantly, by week 32 of the study, 25% of the study patients reached PASI-90 (90% improvement in PASI score from baseline), and 11% reached PASI-100 (100% improvement in PASI score from baseline). Notably, PASI-90 and PASI-100 are the most stringent and difficult to meet clinical endpoints for measuring responses to psoriasis treatments. Moreover, the PASI-90 subset analysis further suggested a higher and significant (*p* = 0.026) piclidenoson response rate of 27% among patients who were naïve to systemic psoriasis therapy compared to those who were pre-treated with such drugs [45]. Analysis of the safety and tolerability of piclidenoson demonstrated that piclidenoson was safe and well-tolerated throughout the 32-week follow-up period in this study. Following the results of phase II/III study, the results of piclidenoson were compared to those of a commercially available oral therapy for psoriasis, apremilast (Otezla; Amgen, Thousand Oaks, CA, USA). The data for apremilast were those from 2 phase III clinical trials, ESTEAM-1 (NCT01194219) and ESTEEM-2 (NCT01232283) (the published research from these two phase III studies, as well as the data included in the label for apremilast) [46,47]. The efficacy for apremilast was found to plateau at 16 weeks with respect to PASI-75 (response rate of approximately 30%), whereas piclidenoson showed no visible plateau at 32 weeks with respect to PASI-75 (response rate of 35.3%). Similarly, piclidenoson was numerically superior to apremilast with respect to PASI-50 and PASI-90 rates at 32 weeks [45].

#### 3.2.3. Piclidenoson in Psoriasis: Phase III Study

A phase III study investigating piclidenoson in psoriasis was designed based on the results of phase II/III study, with apremilast as an active comparator. This phase III psoriasis study named “COMFORT” (NCT03168256) is a multicenter, randomized, double-blind, placebo- and active-controlled (i.e., apremilast) study in adults aged 18–80 years (males and females) with a diagnosis of moderate-to-severe chronic plaque psoriasis. The study enrolled patients at 31 clinical sites in Israel, Moldova, Serbia, Croatia, Poland, Bosnia, Romania, Bulgaria, and Canada. The plan was to include 400 participants and randomize them in a 3:3:3:2 ratio to piclidenoson 2 mg, piclidenoson 3 mg, apremilast 30 mg, or matching placebo tablets. The primary endpoint is the percentage of subjects achieving PASI-75 at week 16 and safety. Secondary endpoints include PASI-50 rate, PGA, and Psoriasis Disability Index (PDI) after 16 weeks of treatment.

Due to the SARS-CoV-2 pandemic, enrollment was paused after 200 patients, and an interim analysis conducted by an independent data monitoring committee (IDMC) was performed after 50% of participants reached the week 16 visit evaluation. In this analysis, piclidenoson was safe and very well tolerated. The findings of the interim analysis led to the recommendation to continue patient enrolment based on both safety and futility. To date, the company completed the enrollment of all 400 patients, and data are expected in the near future.

## 4. Namodenoson: Clinical Development

### 4.1. Namodenson in Hepatocellular Carcinoma (HCC)

HCC is a leading cause of cancer-related death worldwide. More than 600,000 new cases are diagnosed annually worldwide [48]. Most HCC cases are associated with inflammation, usually due to hepatitis B or C virus, and occur primarily in patients with underlying cirrhosis. Thus, when selecting a treatment for HCC, both tumor burden and liver function should be considered. HCC is classified using 2 scoring systems predicting the degree of liver failure in patients with cirrhosis. The first is the Child–Pugh (CP) score, which has a strong prognostic value for HCC patients and is included in all integrated HCC staging systems, and the second is the Barcelona Clinic Liver Cancer (BCLC) system. The CP score is determined using five clinical measures, including total bilirubin, serum albumin, prothrombin time or prolongation of international normalized ratio (INR), ascites, and hepatic encephalopathy. A score of 1, 2, or 3 is given to each measure, with 3 being the most severe. The BCLC includes five prognostic factors: portal vein thrombosis, multifocal tumor, diffuse or massive disease, high alpha-fetoprotein (AFP) levels, and performance status [49].

Advanced HCC is defined as BCLC stage C, or Child–Pugh stages A-C (CPA, CPB, and CPC), and an Eastern Cooperative Oncology Group Performance Score (ECOG PS) of 1–2. Systemic therapy remains the only option for these patient populations. The current therapeutic agents approved for use in advanced HCC can be largely divided into 3 classes, beginning with tyrosine kinase inhibitors (TKI), including sorafenib, lenvatinib, cabozantinib, and regorafenib. The second group includes inhibitors of the vascular endothelial growth factor (VEGF) and the VEGF receptor (VEGFR). The third group includes immune checkpoint inhibitors against either the B7/CTLA-4 or PD-1/ L1 axis, including the anti-PD-1 antibodies, pembrolizumab, and camrelizumab, anti-PD-L1 antibodies, durvalumab, atezolizumab, and avelumab, and the anti-CTLA-4, ipilimumab. Furthermore, several treatment options are currently approved for use in the second-line setting, including nivolumab, nivolumab plus ipilimumab, pembrolizumab, cabozantinib, regorafenib, and ramucirumab [50]. However, these drugs are not given to patients defined as CPB and CPC due to their hepatotoxicity profile.

#### 4.1.1. Namodenoson in HCC: Phase I/II Study

A phase I/II, open-label, dose-escalation study evaluating the safety, tolerability, pharmacokinetics, and pharmacodynamics of orally administered namodenoson in patients with advanced HCC was conducted (NCT00790218). The secondary objectives of the study were to document evidence of the clinical efficacy of namodenoson and to examine the correlation between A3AR expression levels at baseline and patients’ responses to namodenoson.

The study included 18 patients with advanced HCC, where each 6 began dosing with namodenoson at 1 mg, 5 mg, and 25 mg twice daily. Furthermore, 9 subjects have undergone intra-subject dose escalation per protocol. The longest exposure to date is 27.3 months. One patient lived more than 5 years on the drug, and the namodenoson safety experience represented an aggregate of 135.4 months of exposure.

Namodenoson was safe and well-tolerated with no dose-limiting toxicities at any dose level and no changes in vital signs or hematological or chemistry parameters. Most reported adverse events were grade 1 or 2.

Efficacy was evaluated using Response Evaluation Criteria in Solid Tumors (RECIST) v1.0. No complete objective responses (CR) or partial responses (PR) were reported. However, 4 patients experienced stable disease (SD) for at least 4 months. Of these SD patients, one achieved SD for 7 months, and one subject showed complete clinical regression of skin metastases [27]. Out of these four patients, three were administered 25 mg namodenoson and one was administered 12.5 mg. The median overall survival (OS) was 7.8 months (67% of patients progressed on sorafenib, and 28% had CPB disease). The OS of the patients who progressed on sorafenib was 7.0 months, and that of the CPB population was 8.1 months [27]. Namodenoson was found to maintain liver function for 6 months, in distinction from other drugs known to induce hepatotoxicity. Following this study, the Food and Drug Administration (FDA) and the European Medicines Agency (EMA) have granted an ‘orphan drug’ status, and the FDA also granted a ‘fast track’ status for namodenoson in the treatment of advanced HCC.

#### 4.1.2. Namodenoson in HCC: Phase II Study

Following the phase I/II study, a phase II, blinded, randomized, placebo-controlled trial was conducted in patients with advanced HCC and CPB cirrhosis (NCT02128958) [46]. Patients were randomized 2:1 to namodenoson twice daily (25 mg; 50 patients) or placebo (28 patients). The primary endpoint of the OS advantage over the placebo was not met. Median OS was 4.1 and 4.3 months for namodenoson and placebo, respectively (hazard ratio (HR) = 0.82; 95% confidence interval (CI), 0.49 to 1.38; *p* = 0.46). However, a subgroup analysis of patients with CP score of 7, which is the lowest CBP score (34 namodenoson-treated, 22 placebo-treated), showed nonsignificant differences in OS and progression-free survival (PFS) with a numerical advantage for namodenoson (OS: 6.9 vs. 4.3 months; HR = 0.81; 95% CI, 0.45 to 1.43; *p* = 0.46. PFS: 3.5 vs. 1.9 months; HR = 0.89; 95% CI 0.51 to 1.55; *p* = 0.67), and a statistically significant difference in 12-month OS rate (44% vs. 18%, *p* = 0.028). PR was achieved by 3 (9%) of all 50 patients in the namodenoson arm and by none in the placebo arm. Namodenoson was generally well-tolerated. No treatment-related deaths were reported, and no patients withdrew due to toxicity. One grade 3 adverse event was reported in the namodenoson group (hyponatremia, which may also be attributed to cirrhosis) [51]. The positive efficacy signal in HCC CPB7 patients, coupled with the favorable safety profile of namodenoson, supported its continued clinical development in HCC. Consequently, a pivotal phase III study has been designed and approved by the FDA and EMA and is underway (NCT05201404).

### 4.2. Namodenson in NASH

NASH is an advanced form of non-alcoholic fatty liver disease (NAFLD) caused by an accumulation of fat in the liver. This progressive liver disease causes inflammation and damage, leading to scarring of the liver (i.e., cirrhosis) with limited or no therapeutic options [52]. Demonstration of the anti-inflammatory, anti-steatosis and anti-fibrosis effects of namodenoson in the liver in preclinical models led to the clinical development of namodenoson as a potential treatment of NAFLD/NASH [21].

#### Namodenoson in NAFLD/NASH: Phase IIa Study

This phase II blinded, randomized, placebo-controlled trial in patients with NAFLD/NASH randomized patients in a 1:1:1 ratio to namodenoson 12.5 mg twice daily (*n* = 21), namodenoson 25 mg twice daily (*n* = 19) or placebo (*n* = 20) for 12 weeks and patients were followed up for a total of 16 weeks (NCT02927314) [53]. The randomization was stratified by the presence or absence of type II diabetes mellitus. The anti-inflammatory effect of namodenoson was demonstrated by a decrease from baseline in mean serum alanine aminotransferase (ALT) and aspartate aminotransferase (AST) levels as well as by a statistically significantly higher proportion of patients in the 25 mg arm vs. the placebo arm, achieving normalization of serum ALT levels at week 16 (37% vs. 10%, *p* = 0.038). Adiponectin levels were increased in the namodenoson-treated patients, further corroborating the anti-inflammatory effect of namodenoson treatment. Treatment with namodenoson decreased fibrosis-4 (FIB-4), and FibroScan-AST (FAST) scores that are non-invasive biomarkers used to identify advanced fibrosis. FIB-4 scores demonstrated a statistically significant decrease from screening to week 12 in the namodenoson 25 mg dose group (*p* = 0.011), demonstrating a possible decrease in the degree of liver fibrosis in patients treated with namodenoson 25 mg. The within-group analysis demonstrated a statistically significant reduction in FAST scores from screening to week 12 in the 25 mg dose group (*p* = 0.002). In contrast, in the placebo group, this reduction was not statistically significant [53]. A linear decrease in body weight was observed in both the namodenoson 12.5 mg and namodenoson 25 mg treatment groups throughout the study, with a greater decrease observed in the namodenoson 25 mg group (the loss of weight in the treatment groups was not statistically significantly different from that in the placebo arm). Treatment with namodenoson was well tolerated with no treatment-related emergent severe adverse events, no drug-related treatment withdrawals, or hepatotoxicity [53]. These positive data led to the design and conduction of a phase IIb study (NCT04697810), which is currently ongoing.

## 5. Conclusions

The current report describes the clinical development of two drugs, piclidenoson and namodenoson, both of which are A3AR agonists. Based on both the safety and efficacy of the phase II study data, the piclidenoson development program is progressing toward phase III to treat psoriasis, and the namodenoson program is progressing toward phase III to treat advanced HCC and phase IIb for the treatment of NASH. The uniqueness of these drug candidates stems from their ability to bind specifically to A3AR, which is highly expressed in pathological but not normal cells. This specificity results in a favorable safety profile found consistently throughout their clinical development. The mechanism of action that has been shown in preclinical studies entails the modulation of key signaling proteins as the basis for drug efficacy. The data thus far suggest that piclidenoson and namodenoson could potentially be the first A3AR agonists to achieve FDA approval and provide clinicians with new oral and safe drugs in the arsenal for fighting psoriasis (piclidenoson), HCC and NASH (namodenoson).

## Data Availability

Not applicable.
